# Disinfectant Susceptibility of Third-Generation-Cephalosporin/Carbapenem-Resistant Gram-Negative Bacteria Isolated from the Oral Cavity of Residents of Long-Term-Care Facilities

**DOI:** 10.1128/aem.01712-22

**Published:** 2022-12-14

**Authors:** Azusa Haruta, Miki Kawada-Matsuo, Mi Nguyen-Tra Le, Mineka Yoshikawa, Toshiki Kajihara, Koji Yahara, Norikazu Kitamura, Shoko Kutsuno, Chika Arai, Maho Takeuchi, Yo Sugawara, Junzo Hisatsune, Kazuhiro Tsuga, Hiroki Ohge, Motoyuki Sugai, Hitoshi Komatsuzawa

**Affiliations:** a Department of Advanced Prosthodontics, Hiroshima University Graduate School of Biomedical and Health Sciences, Hiroshima, Japan; b Department of Bacteriology, Hiroshima University Graduate School of Biomedical and Health Sciences, Hiroshima, Japan; c Project Research Center for Nosocomial Infectious Diseases, Hiroshima University, Hiroshima, Japan; d Antimicrobial Resistance Research Center, National Institute of Infectious Diseases, Tokyo, Japan; e Department of Infectious Diseases, Hiroshima University Hospital, Hiroshima, Japan; Centers for Disease Control and Prevention

**Keywords:** antimicrobial-resistant bacteria, disinfectants, elderly, long-term-care facility, oral microbiology, susceptibility testing

## Abstract

We have recently reported the isolation of third-generation-cephalosporin-resistant Gram-negative bacteria from the oral cavity of residents of a long-term-care facility (LTCF). Since disinfectants are often used in the oral cavity, it is important to investigate the disinfectant susceptibility of oral bacteria. Here, we evaluated the susceptibilities of Gram-negative antimicrobial-resistant bacteria (GN-ARB), including Pseudomonas, Acinetobacter, and *Enterobacteriaceae*, obtained from the oral cavity of residents of LTCFs to povidone-iodine (PVPI), cetylpyridinium chloride (CPC), benzalkonium chloride (BZK), and chlorhexidine chloride (CHX). We also evaluated the susceptibilities of isolates from the rectum to the same agents to compare the susceptibility profiles of oral and rectal isolates. Next, we investigated the relationship between their susceptibility and disinfectant resistance genes delineated by whole-genome sequencing of the isolates. Additionally, we evaluated the correlation between disinfectant-resistant GN-ARB and clinical information. In oral GN-ARB, the MIC of PVPI showed almost identical values across isolates, while the MICs of CPC, BZK, and CHX showed a wide range of variation among species/strains. In particular, Pseudomonas aeruginosa exhibited high-level resistance to CPC and BZK. The disinfectant susceptibility of rectal GN-ARB showed a tendency similar to that of oral GN-ARB. The presence of *qacE*Δ*1* was correlated with CPC/BZK resistance in P. aeruginosa, while other species exhibited no correlation between *qacE*Δ*1* and resistance. Multiple analyses showed the correlation between the presence of CPC-resistant bacteria in the oral cavity and tube feeding. In conclusion, we found that some oral GN-ARB isolates showed resistance to not only antibiotics but also disinfectants.

**IMPORTANCE** Antibiotic-resistant bacteria (ARB) are becoming a serious concern worldwide. We previously reported the isolation of third-generation-cephalosporin-resistant Gram-negative bacteria from the oral cavity of residents of a long-term-care facility (LTCF). To prevent infection with ARB in hospitals and eldercare facilities, we must pay more attention to the use of not only antibiotics but also disinfectants. However, the effect of disinfectants on ARB is unclear. In this study, we evaluated the susceptibility of Gram-negative ARB (GN-ARB) from the oral cavity of residents of LTCFs to some disinfectants that are often used for the oral cavity; we found that some isolates showed resistance to several disinfectants. This is the first comprehensive analysis of the disinfectant susceptibility of oral GN-ARB. These results provide some important information for infection control and suggest that disinfectants should be applied carefully.

## INTRODUCTION

Antimicrobial-resistant bacteria (ARB) are a worldwide problem. Among ARB, Gram-negative bacteria, including third-generation-cephalosporin- and/or carbapenem-resistant *Enterobacteriaceae*, Pseudomonas, and Acinetobacter, have been a recent global concern. These bacteria can be isolated from various sites of the human body, such as normal sites (rectum and skin) and infected sites (blood and urine), and the hospital environment. However, there have been few reports regarding oral Gram-negative ARB (GN-ARB). Recently, Le et al. reported the isolation of GN-ARB from the oral cavity of residents of a long-term-care facility (LTCF) (1). We also reported the isolation of several oral GN-ARB species, including Acinetobacter baumannii, Pseudomonas aeruginosa, and Escherichia coli, in 6 LTCFs (2). The frequencies of isolation of oral and rectal GN-ARB were 17.4% and 54.5% of residents in 6 LTCFs, respectively. Among GN-ARB isolates, extended-spectrum-β-lactamase (ESBL)-producing *Enterobacterales* and P. aeruginosa were detected in 42.7% and 2.8% of rectal swabs and 5.6% and 3.4% of oral swabs, respectively (2).

Pneumonia is one of the main causes of death, especially among older adults (3). Aspiration pneumonia is a particularly common type of pneumonia in the elderly population (4). Aspiration pneumonia occurs when oropharyngeal contents containing pathogenic bacteria accidentally enter the respiratory trachea (3). Older adults requiring care experience frequent dysphagia due to cerebrovascular diseases or other causes; consequently, they are at a high risk for aspiration (3, 4). Many pathogenic bacteria, including Klebsiella pneumoniae, Enterobacter, P. aeruginosa, E. coli, and Proteus mirabilis, were identified as being the major causes of respiratory infectious diseases (5, 6). Additionally, multidrug-resistant Gram-negative bacteria that cause aspiration pneumonia have been reported (7). GN-ARB have been reported to be pathogens that cause hospital-acquired pneumonia and community-acquired pneumonia (7–9). Several GN-ARB are considered to be causative pathogens of aspiration pneumonia that responds poorly to antimicrobial treatment (5, 7).

It has been shown that oral care reduces the incidence of pneumonia in older adults, and it is important for nursing home residents (10–12). Mouthwashes are sometimes used during oral care (9, 10). Mouthwashes containing disinfectants, such as povidone-iodine (PVPI), quaternary ammonium compounds (QACs) such as cetylpyridinium chloride (CPC) and benzalkonium chloride (BZK), and chlorhexidine (CHX), are widely used as common medical and consumer products (13, 14). For example, before dental surgical treatment and cleaning of periodontal pockets, disinfectants are usually used. Therefore, if disinfectant-resistant GN-ARB are present in the oral cavity, disinfectants should be used carefully. However, the degree of resistance (R) of oral GN-ARB to disinfectants is not clear.

In this study, we examined the susceptibility (S) of GN-ARB to disinfectants. Next, we aimed to identify the relationship between the genes responsible for resistance to disinfectants and the actual susceptibility of the bacteria to disinfectants. Additionally, we evaluated the correlation of GN-ARB with clinical information.

## RESULTS

### Isolation of third-generation-cephalosporin- or carbapenem-resistant Gram-negative bacteria.

The isolates used in this study are shown in Table 1. We used 80 isolates obtained from a previous study (2) and 47 isolates specific to this study, although both sets of isolates were obtained from the same 178 residents in LTCFs (see Fig. S1 in the supplemental material). Among these isolates, we used 32 that were previously isolated from the oral cavity of 27 residents, and we performed whole-genome sequencing of these bacteria (2). We also isolated 20 carbapenem-resistant GN-ARB from the oral cavity of 18 residents and 70 isolates from the rectum of 61 residents. Since our focus was the susceptibility of oral isolates to disinfectants, we selected all 52 oral isolates, including 32 isolates selected by third-generation-cephalosporin resistance and 20 isolates selected by carbapenem resistance. To compare the susceptibilities of oral and rectal isolates, we selected a total of 75 isolates, comprising 48 selected by third-generation-cephalosporin resistance and 27 selected by carbapenem resistance.

**TABLE 1:** Numbers of cephalosporin/carbapenem-resistant isolates by bacterial species from the oral cavity and rectum

Organism	No. of isolates			
	Oral	Rectal + oral^*a*^		Rectal^*b*^
		Same^*c*^	Different^*d*^	
Acinetobacter				
A. baumannii	5	1	0	2
A. baylyi	1	0	0	0
*A. lactucae*	1	0	0	0
A. nosocomialis	5	0	0	0
A. pittii	2	0	0	0
*A. seifertii*	2	0	0	0
*A. ursingii*	2	0	0	0
Enterobacter				
E. bugandensis	1	2	0	0
E. cloacae	0	0	1	0
*E. hormaechei*	4	1	1	2
*E. roggenkampii*	0	0	1	0
Enterobacter spp. (unknown)	1	0	0	0
Escherichia coli	5	3	17	19
Pseudomonas				
P. aeruginosa	8	1	1	13
*P. citronellolis*	4	0	0	0
*P. fulva*	2	0	0	0
*P. nitroreducens*	2	0	0	0
Proteus mirabilis	2	1	1	4
Klebsiella pneumoniae	0	0	2	2
Achromobacter xylosoxidans	2	0	0	0
Others				
Pandoraea apista	1	0	0	0
Pantoea septica	1	0	0	0
Stenotrophomonas maltophilia	1	0	0	0

a Rectal isolates from subjects with GN-ARB from the oral cavity.

b Rectal isolates from subjects without GN-ARB from the oral cavity.

c Rectal isolates of the same species as those of the oral isolates from the same subjects.

d Rectal isolates of different species than those of the oral isolates from the same subjects.

### MICs of disinfectants.

Table 2 shows the susceptibilities of cephalosporin- and carbapenem-resistant Gram-negative bacteria isolated from the oral cavity and rectal cavity to 4 disinfectants. Among the bacterial strains used in this study, the MIC of PVPI ranged from 1,094 to 4,375 μg/mL (Table 2; Table S1a). Unlike the MIC of PVPI, the MICs of CPC, BZK, and CHX varied among strains, showing a wide range of MIC values from low to high. The MIC of CPC ranged from 2.5 to 5,120 μg/mL. The MICs of CPC in 22 P. aeruginosa isolates (7 out of 8 oral isolates and 15 rectal isolates) and 2 oral Achromobacter xylosoxidans isolates were quite high, showing a range from 160 to 5,120 and 160 μg/mL, respectively (Table 2; Table S1b). Acinetobacter tended to show a lower MIC of CPC (2.5 to 20 μg/mL) than other bacteria. The MIC of BZK ranged from 2.5 to 640 μg/mL overall. The MIC variation of BZK showed a tendency similar to that of CPC. The MICs of BZK in 19 P. aeruginosa and 2 A. xylosoxidans isolates were high (ranging from 40 to 640 and from 40 to 80 μg/mL, respectively) (Table 2; Table S1c). In addition, the MICs of BZK in some P. mirabilis and Acinetobacter seifertii isolates were high (20 to 40 and 5 to 40 μg/mL, respectively). Overall, the MIC of CHX ranged from 4 to 256 μg/mL. Among Acinetobacter isolates, A. nosocomialis showed a higher MIC of CHX (16 to 256 μg/mL) than the other Acinetobacter isolates, except for 3 A. baumannii isolates and 1 A seifertii isolate (16 to 32 and 16 μg/mL, respectively) (Table 2; Table S1d). All P. mirabilis and some K. pneumoniae isolates (2 rectal isolates from a person who also provided oral isolates) showed high MICs of CHX (32 to 128 μg/mL), while some P. aeruginosa isolates (16/23 isolates) and all Enterobacter isolates, except for 1 rectally isolated E. roggenkampii isolate, showed high MICs (16 to 64 μg/mL). In the comparison of MICs between oral and rectal isolates, there were no significant differences.

**TABLE 2:** Susceptibility of cephalosporin/carbapenem-resistant oral/rectal isolates to disinfectants by bacterial species

Organism	Type of isolate	No. of isolates	PVPI		CPC		BZK		CHX	
			MIC_50_	MIC range (μg/mL)	MIC_50_	MIC range (μg/mL)	MIC_50_	MIC range (μg/mL)	MIC_50_	MIC range (μg/mL)
Acinetobacter										
A. baumannii	Oral	5	4,375	2,188–4,375	5	2.5–5	2.5	2.5–5	4	4–32
	Rectal + oral^*a*^	1		2,188		2.5		2.5		8
	Rectal (only)^*b*^	2	4,375	4,375	2.5	2.5	5	5	24	16–32
	ATCC 19606^*c*^			1,094		2.5		5		64
	ATCC 17978^*c*^			2,188		5		5		32
A. baylyi	Oral	1		2,188		2.5		5		4
*A. lactucae*	Oral	1		1,094		2.5		2.5		4
A. nosocomialis	Oral	5	2,188	1,094–2,188	5	2.5–20	5	5–10	64	16–256
A. pittii	Oral	2	1,094	1,094	3.8	2.5–5	2.5	2.5	4	4
	ATCC 19004^*c*^			1,094		2.5		2.5		8
*A. seifertii*	Oral	2	1,094	1,094	2.5	2.5	22.5	5–40	10	4–16
*A. ursingii*	Oral	2	1,094	1,094	2.5	2.5	2.5	2.5	4	4
Achromobacter xylosoxidans	Oral	2	2,188	2,188	160	160	60	40–80	4	4
Enterobacter										
*E. bugandensis*	Oral	1		4,375		10		20		32
	Rectal + oral^*a*^	2	4,375	4,375	30	20–40	20	20	32	32
E. cloacae	Rectal + oral^*a*^	1		4,375		10		20		64
*E. hormaechei*	Oral	4	3,282	2,188–4,375	10	5–10	10	5–20	24	16–32
	Rectal + oral^*a*^	2	4,375	4,375	10	10	10	10	24	16–32
	Rectal (only)^*b*^	2	4,375	4,375	10	10	10	10	24	16–32
*E. roggenkampii*	Rectal + oral^*a*^	1		4,375		10		20		8
Enterobacter spp. (unknown)	Oral	1		4,375		40		20		16
Escherichia coli	Oral	5	4,375	2,188–4,375	3.8	2.5–40	10	5–40	4	4–8
	Rectal + oral^*a*^	20	4,375	2,188–4,375	2.5	2.5–5	10	5–10	8	4–16
	Rectal (only)^*b*^	19	4,375	1,094–4,375	2.5	2.5–10	5	5–20	4	4–128
	K-12^*c*^			2,188		5		10		64
	ATCC 25922^*c*^			2,188		5		5		8
Pseudomonas										
P. aeruginosa	Oral	8	4,375	2,188–4,375	960	2.5–5,120	60	2.5–640	10	4–64
	Rectal + oral^*a*^	2	4,375	4,375	1,600	640–2,560	90	20–160	12	8–16
	Rectal (only)^*b*^	13	4,375	2,188–4,375	640	160–5,120	40	10–640	16	4–32
	PAO1^*c*^			4,375		320		40		64
	ATCC 27853^*c*^			4,375		320		40		16
*P. citronellolis*	Oral	4	4,375	4,375	20	10–20	20	20	4	4
*P. fulva*	Oral	2	3,282	2,188–4,375	10	10	7.5	5–10	4	4
*P. nitroreducens*	Oral	2	2,735	1,094–4,375	10	10	20	20	4	4
Proteus mirabilis	Oral	2	4,375	4,375	15	10–20	40	40	64	64
	Rectal + oral^*a*^	2	4,375	4,375	15	10–20	30	20–40	96	64–128
	Rectal (only)^*b*^	4	4,375	4,375	15	10–20	40	20–40	128	64–128
										
Klebsiella pneumoniae	Rectal + oral^*a*^	2	4,375	4,375	7.5	5–10	7.5	5–10	80	32–128
	Rectal (only)^*b*^	2	4,375	4,375	5	5	10	10	6	4–8
	ATCC BAA-1706^*c*^			2,188		5		10		64
Others										
Pandoraea apista	Oral	1		4,375		10		10		4
Pantoea septica	Oral	1		1,094		10		5		4
S. maltophilia	Oral	1		1,094		5		20		4

a Rectal isolates from subjects with GN-ARB from the oral cavity.

b Rectal isolates from subjects without GN-ARB from the oral cavity.

c Standard strain.

Next, we evaluated the susceptibilities of standard strains, including A. baumannii (2 strains), Acinetobacter pittii (1 strain), E. coli (2 strains), Enterobacter cloacae (1 strain), and P. aeruginosa (2 strains). Most of the four disinfectants had low MICs against all of these strains, similar to their low MIC values against the oral and rectal isolates. The strains with high MICs for these disinfectants were as follows: the MICs of E. coli K-12, P. aeruginosa PAO1, A. baumannii ATCC 19606, and K. pneumoniae ATCC BAA-1706 for CHX were 64 μg/mL.

In addition, the MIC values of the disinfectants against the isolates were compared to the concentrations at which these disinfectants are commonly used in the oral cavity. The ranges for the concentrations of PVPI, CPC, BZK, and CHX were 2,188 to 4,375 μg/mL, 400 to 800 μg/mL, 80 to 800 μg/mL, and 1,229 to 2,048 μg/mL, respectively (Table S2). The MIC values of PVPI and CHX against all isolates were lower than the concentrations used in the oral cavity, although the MIC values of CHX were variable among the isolates. Regarding CPC and BZK, their MICs against some isolates were higher than the concentrations used in the oral cavity.

### Comparison of the strains isolated from the oral and rectal regions of the same participant.

Among 38 participants with resistant bacteria found in the oral cavity, 9 participants had the same bacterial species isolated from the oral cavity and the rectum. By comparing the sequence types (STs) of the oral and rectal isolates, we observed that six individuals had isolates with the same ST in both the oral and rectal regions, while the isolates from the oral and rectal sites in one individual were confirmed to have different STs (Table 3). In addition, we compared the susceptibilities of oral and rectal isolates to disinfectants and antibacterial agents. Any two isolates exhibiting the same ST showed approximately the same degree of susceptibility to these agents, within a 2-fold difference, excluding 2 isolates (K0355 and K0307). Two isolates exhibiting different STs showed different degrees of susceptibility to some agents in one person (K0240 and K0171). As for the isolates without an ST, isolates of Enterobacter bugandensis from the two sites showed similar patterns of susceptibility to antibacterial agents, but CPC susceptibility showed a 4-fold difference. Isolates of P. mirabilis from the two sites showed similar patterns of susceptibility to both agents.

**TABLE 3:** Comparison of the characteristics of oral and rectal isolates from a single subject

Species	Isolate	Site^*a*^	ST	No. of SNPs (no. of synonymous mutations)^*b*^	MIC of disinfectant (μg/mL)				Susceptibility to antibacterial agent (μg/mL)^*c*^									
					PVPI	CPC	BZK	CHX	CTX	CTRX	CAZ	MEM	IPM	ATM	CFPM	CMZ	PIP-TAZ	LVX
*E. hormaechei*	K0355	O	133	57 (28)	2,188	5	5	32	>2	>2	2	≤0.25	≤0.5	>8	2	>32	ND	≤0.12
	K0307	R	133		4,375	10	10	16	>2	>2	>8	≤0.25	≤0.5	>8	>16	>32	≤4	1
		
*E. hormaechei*	K0360	O	662	7 (1)	4,375	10	20	16	1	≤0.5	≤1	≤0.25	≤0.5	≤1	≤1	>32	≤4	≤0.12
	K0294	R	662		4,375	10	10	32	1	1	≤1	≤0.25	1	≤1	≤1	>32	≤4	≤0.12

P. aeruginosa	K0240	O	244	ND	4,375	1,280	40	64	ND	ND	2	≤0.5	2	4	2	ND	16	≤0.5
	K0171	R	664	ND	4,375	640	20	16	ND	ND	16	1	1	>16	8	ND	64	≤0.5

A. baumannii	K0233	O	354	4 (0)	2,188	5	2.5	4	>2	>2	2	0.5	≤0.5	8	2	>32	ND	1
	K0151	R	354		2,188	2.5	2.5	8	>2	>2	4	0.5	≤0.5	>8	4	>32	ND	1

*E. bugandensis*	K0356	O		24 (2)	4,375	10	20	32	>2	>2	4	≤0.25	1	2	≤1	>32	≤4	≤0.12
	K0287	R			4,375	40	20	32	>2	>2	4	≤0.25	1	4	≤1	>32	8	≤0.12

E. coli	K0191	O	131	7 (0)	2,188	2.5	10	8	>2	>2	>8	≤0.25	≤0.5	>8	>16	≤4	≤4	>4
	K0052	R	131		4,375	5	10	4	>2	>2	>8	≤0.25	≤0.5	>8	>16	≤4	≤4	>4

E. coli	K0235	O	91	76 (27)	4,375	5	40	4	>2	>2	8	≤0.25	≤0.5	>8	>16	≤4	≤4	≤0.12
	K0165	R	91		4,375	5	10	4	>2	>2	>8	≤0.25	≤0.5	>8	>16	16	16	≤0.12

E. coli	K0350	O	69	25 (3)	4,375	5	10	4	>2	>2	8	≤0.25	≤0.5	>8	>16	≤4	≤4	≤0.12
	K0289	R	69		4,375	2.5	10	4	>2	>2	4	≤0.25	≤0.5	>8	>16	≤4	≤4	≤0.12

P. mirabilis	K0224	O		24 (5)	4,375	20	40	64	>2	>2	≤1	≤0.25	ND	4	>16	≤4	≤4	1
	K0124	R			4,375	20	20	128	>2	>2	≤1	≤0.25	ND	≤1	16	≤4	≤4	1

a O, orally isolated; R, rectally isolated.

b The number of synonymous mutations is indicated in parentheses.

c CTX, cefotaxime; CTRX, ceftriaxone; CAZ, ceftazidime; MEM, meropenem; IPM, imipenem; ATM, aztreonam; CFPM, cefepime; CMZ, cefmetazole; PIP-TAZ, piperacillin-tazobactam; LVX, levofloxacin; ND, not determined.

Next, single nucleotide polymorphism (SNP) analysis was performed on the isolates with the same ST or the same pattern of susceptibility to antibiotics (Table 3). The numbers of SNPs in each set were different. Six of eight pairs had fewer than 30 SNPs, while two pairs had more, with 76 and 57 SNPs (Table 3). The SNPs were found to be located in intergenic regions and open reading frames (ORFs); both synonymous and nonsynonymous mutations were represented (Table S3). Upon comparing the SNP sites between 2 sets of Enterobacter hormaechei or 3 sets of E. coli isolates, we found no common sites (Table S3).

### Genes responsible for resistance to disinfectants.

**FIG 1:**
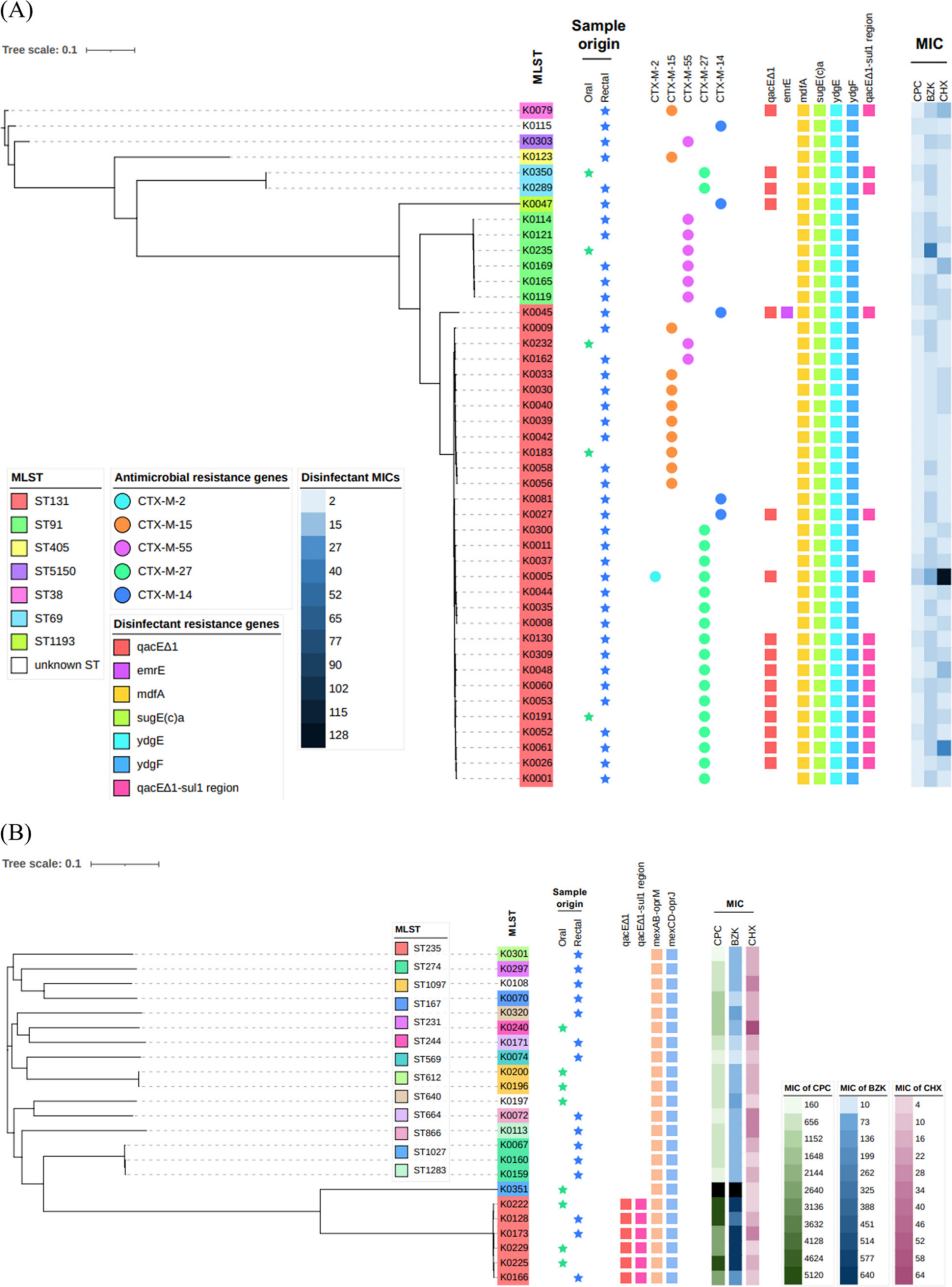

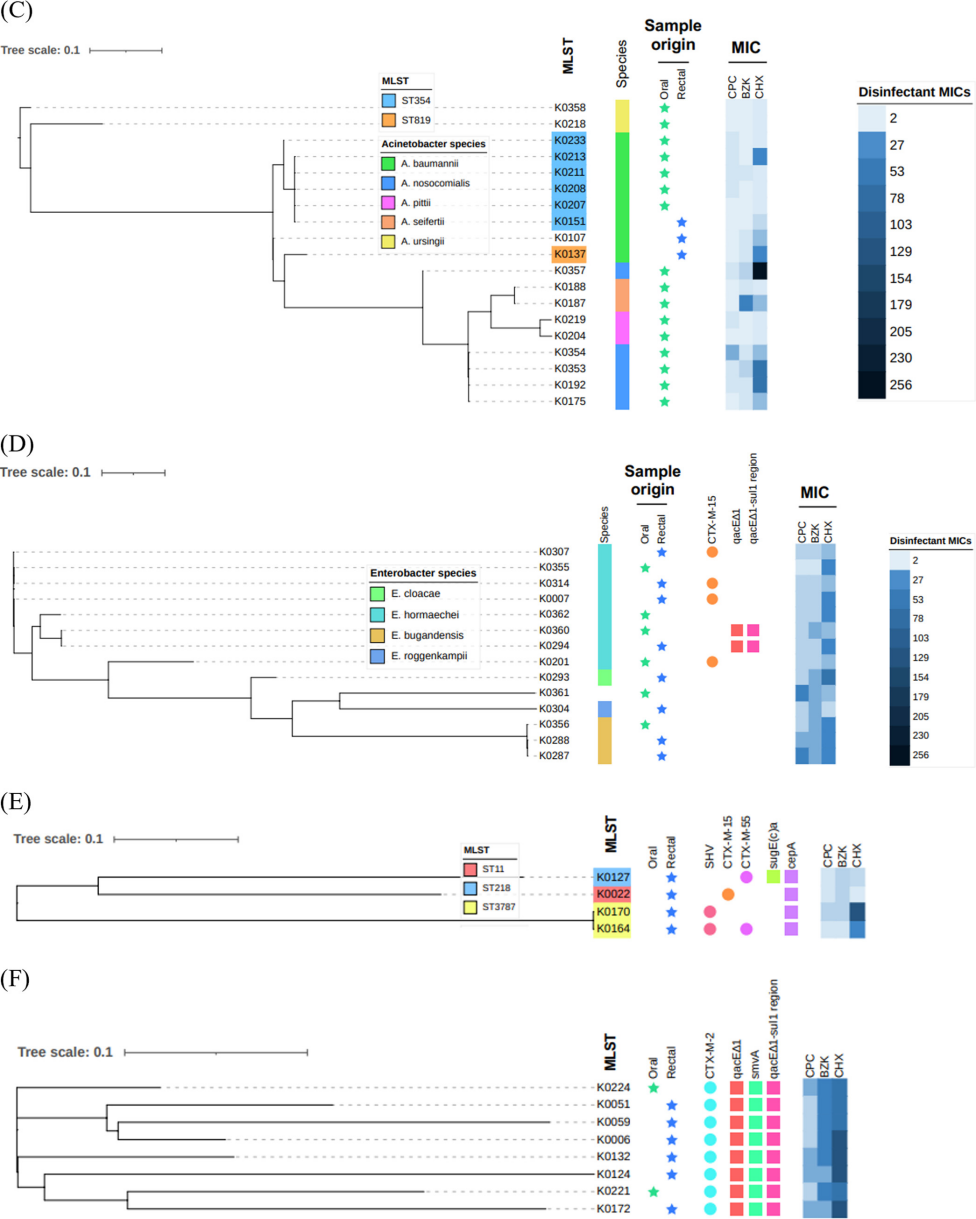
Phylogenetic analysis and relationships between resistance genes and MICs of disinfectants. (A) E. coli; (B) P. aeruginosa; (C) Acinetobacter spp.; (D) Enterobacter spp.; (E) K. pneumoniae; (F) P. mirabilis. In the MLST analysis results, the background color indicates the strain ST. ESBL genes and disinfectant genes are indicated with circles and squares, respectively. The MIC values of disinfectants are indicated by the color shade, as shown in the key, and they differed according to the MIC of each species.

Twenty-two disinfectant resistance genes were analyzed in all examined isolates. In E. coli, all oral and rectal isolates possessed *mdfA*, *sugE(c)*, and *ydgEF*, while *qacE*Δ*1* was found in 2 of 5 oral isolates and 14 of 39 rectal isolates (8 of 20 rectal isolates from subjects with oral GN-ARB and 6 of 19 rectal isolates from subjects without oral GN-ARB) (Table 4). Among 14 isolates of the genus Enterobacter, only 2 E. hormaechei isolates from one person possessed *qacE*Δ*1* (Table 4 and Fig. 1). For the genus Pseudomonas, only P. aeruginosa possessed *qacE*Δ*1*: 3 of 16 oral isolates and 3 of 15 rectal isolates (1 of 2 rectal isolates from subjects with oral GN-ARB and 4 of 4 rectal isolates from subjects without oral GN-ARB) (Table 4 and Fig. 1). All 4 P. mirabilis isolates possessed *qacE*Δ*1* and *smvA*. Two K. pneumoniae isolates from the rectum possessed *cepA*. Acinetobacter and all other bacteria lacked disinfectant resistance genes. Furthermore, none of the other 11 genes examined in this study were found in any isolates.

**TABLE 4:** GN-ARB ESBL-producing genes and disinfectant resistance genes

Genus	Type of isolate	No. of isolates	No. of isolates carrying:															
			ESBL gene						Disinfectant resistance gene									
			SHV	CTX-M-15	CTX-M-55	CTX-M-2	CTX-M-14	CTX-M-27	*qacA–J*	*qacE*Δ*1*	*mdfA*	*sugE(c)*	*ydgEF*	*emrE*	*smvA*	*cepA*	*mexAB-oprM*	*mexCD-oprJ*
Acinetobacter	Oral	18	0	0	0	0	0	0	0	0	0	0	0	0	0	0	0	0
	Rectal + oral^*a*^	1	0	0	0	0	0	0	0	0	0	0	0	0	0	0	0	0
	Rectal (only)^*b*^	2	0	0	0	0	0	0	0	0	0	0	0	0	0	0	0	0

Escherichia coli	Oral	5	0	1	2	0	0	1	0	2	5	5	5	0	0	0	0	0
	Rectal + oral^*a*^	20	0	3	6	0	2	7	0	8	20	20	20	0	0	0	0	0
	Rectal (only)^*b*^	19	0	7	1	1	3	8	0	6	19	19	19	1	0	0	0	0

Enterobacter	Oral	6	0	1	0	0	0	0	0	1	0	0	0	0	0	0	0	0
	Rectal + oral^*a*^	6	0	1	0	0	0	0	0	1	0	0	0	0	0	0	0	0
	Rectal (only)^*b*^	2	0	2	0	0	0	0	0	0	0	0	0	0	0	0	0	0

Pseudomonas	Oral	16	0	0	0	0	0	0	0	3	0	0	0	0	0	0	8	8
	Rectal + oral^*a*^	2	0	0	0	0	0	0	0	1	0	0	0	0	0	0	2	2
	Rectal (only)^*b*^	13	0	0	0	0	0	0	0	2	0	0	0	0	0	0	13	13

Proteus	Oral	2	0	0	0	2	0	0	0	2	0	0	0	0	2	0	0	0
	Rectal + oral^*a*^	2	0	0	0	2	0	0	0	2	0	0	0	0	2	0	0	0
	Rectal (only)^*b*^	4	0	0	0	4	0	0	0	4	0	0	0	0	4	0	0	0

Klebsiella pneumoniae	Oral	0	0	0	0	0	0	0	0	0	0	0	0	0	0	0	0	0
	Rectal + oral^*a*^	2	2	0	1	0	0	0	0	0	0	0	0	0	0	2	0	0
	Rectal (only)^*b*^	2	0	1	1	0	0	0	0	0	0	1	0	0	0	2	0	0

Others	Oral	5	0	0	0	0	0	0	0	0	0	0	0	0	0	0	0	0
	Rectal + oral^*a*^	0	0	0	0	0	0	0	0	0	0	0	0	0	0	0	0	0
	Rectal (only)^*b*^	0	0	0	0	0	0	0	0	0	0	0	0	0	0	0	0	0

a Rectal isolates from subjects with GN-ARB from the oral cavity.

b Rectal isolates from subjects without GN-ARB from the oral cavity.

Next, we investigated the relationship between disinfectant susceptibility and the presence of resistance genes (Fig. 1). In E. coli, the MICs of 4 disinfectants against 16 *qacE*Δ*1*-positive isolates were similar to their MICs against 28 *qacE*Δ*1*-negative isolates. All E. coli isolates possessed *mdfA*, *sugE(c)*, and *ydgEF*; therefore, we did not compare the susceptibilities between gene-positive and gene-negative isolates. Two *E. hormaechei* isolates with *qacE*Δ*1* showed approximately the same susceptibilities to the 4 disinfectants as those of the isolates without *qacE*Δ*1*. P. aeruginosa isolates with *qacE*Δ*1* showed significantly lower susceptibilities to CPC and BZK than the isolates without *qacE*Δ*1* (*P* < 0.001 by a Wilcoxon rank sum test) (Table S4). P. mirabilis isolates with *qacE*Δ*1* and *smvA* showed lower levels of susceptibility to CHX, but not CPC and BZK, than the other isolates (Table 1). Two K. pneumoniae isolates from only the rectum possessed *cepA* and showed lower levels of susceptibility to CHX than the other isolates.

### Genetic map of the integron 1 region, including *qacE*Δ*1*, in 4 species.

**FIG 2:**
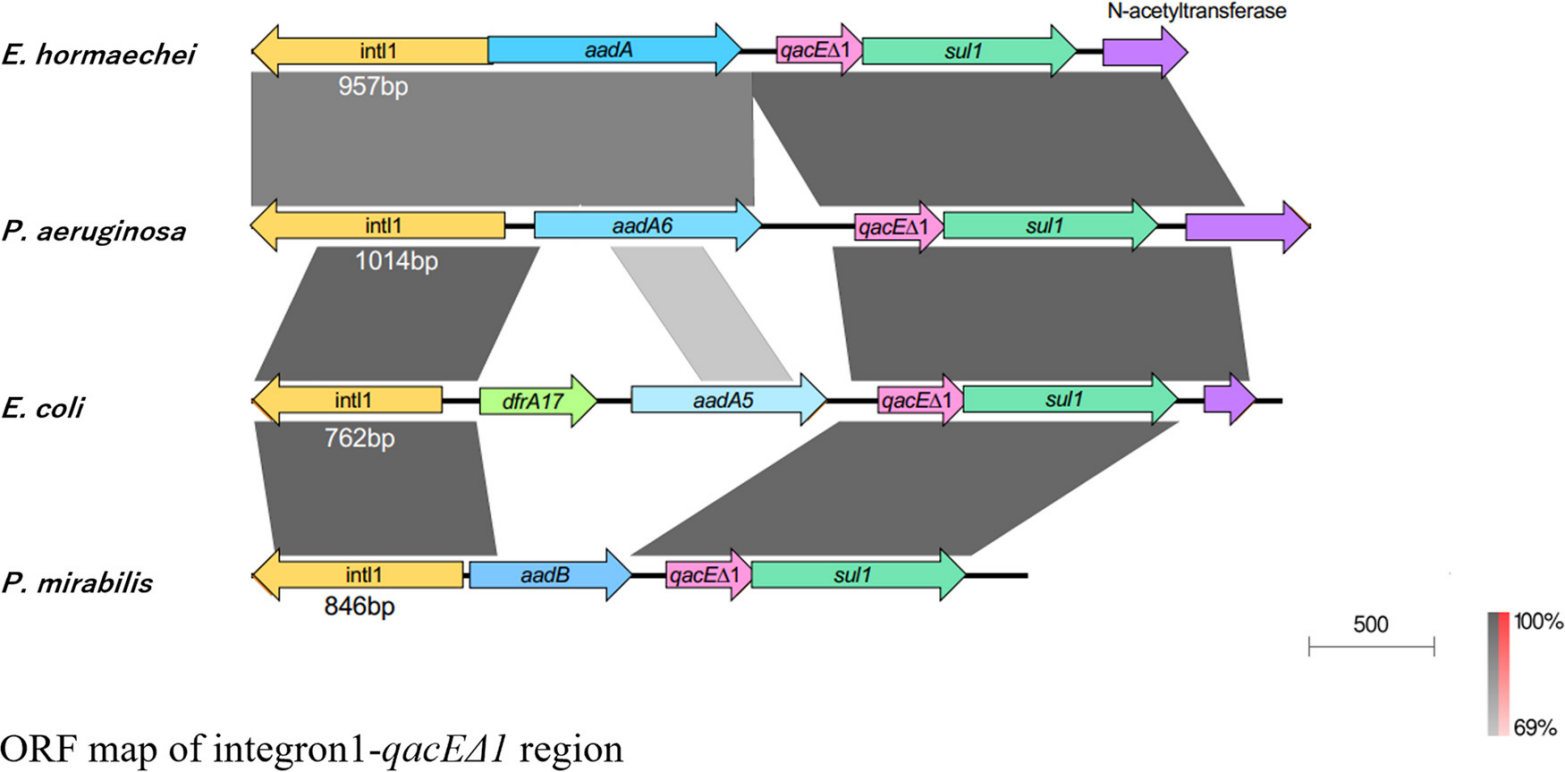
ORF map of the integron 1-*qacE*Δ*1* region. One isolate per species with the *qacE*Δ*1* gene was used as a representative: *E*. *hormaechei* K0360, P. aeruginosa K0222, E. coli K0026, and P. mirabilis K0006.

Since we observed a relationship between *qacE*Δ*1* and CPC/BZK resistance in P. aeruginosa but not E. coli, *E. hormaechei*, or P. mirabilis, we compared the genetic maps of *qacE*Δ*1*-positive P. aeruginosa, E. coli, *E. hormaechei*, and P. mirabilis isolates (Fig. 2). We found that *qacE*Δ*1*, in addition to the gene for aminoglycoside resistance, was located between the *intl1* and *sul1* (sulfonamide resistance) genes in four different species. The nucleotide sequences of *qacE*Δ*1* and *sul1* were quite similar among species. Those of aminoglycoside resistance factors and the integrase 1 gene were different among species but showed similarity among strains. In P. aeruginosa, *E. hormaechei*, and P. mirabilis, the *int1* region contained 4 genes, namely, the integrase 1 gene, the aminoglycoside resistance gene, *qacE*Δ*1*, and *sul1*, while five genes (*int1*, *dfrA17* [dihydrofolate reductase], *aadA5*, *qacE*Δ*1*, and *sul1*) were found between *intl1* and *sul1* in E. coli.

### Relationship of participant conditions with susceptibility to disinfectants.

We used the criteria regarding the concentrations for oral application; the results are shown in Table 5. We found that CPC resistance or intermediate resistance (I) was significantly related to tube feeding (*P* = 0.0088). No factors were significantly associated with BZK resistance or intermediate resistance. PVPI resistance or intermediate resistance showed a significant relationship with the use of antibiotics within the previous 6 months (*P* = 0.045). Furthermore, to confirm whether these items were associated with disinfectant resistance regardless of other factors, we performed multiple logistic regression analysis on each item after adjusting for covariates; this analysis showed that resistance to CPC was associated with tube feeding (odds ratio [OR], 22.6 [95% confidence interval {CI}, 1.82 to 281]; *P* = 0.015). All isolates showed MIC values lower than the concentrations used for oral application; therefore, the relationship with CHX was excluded.

**TABLE 5:** Clinical characteristics of patients and risk factors associated with oral isolates by susceptibility to disinfectants (concentration of use in the oral cavity)^*a*^

Variable	PVPI					CPC					BZK				
	No. (%) of subjects^*b*^		Univariate analysis		*P* value^*c*^	No. (%) of subjects^*b*^		Univariate analysis		*P* value^*c*^	No. (%) of subjects^*b*^		Univariate analysis		*P* value^*c*^
	Group 1 (*n* = 18)	Group 2 (*n* = 12)	OR	95% CI		Group 1 (*n* = 6)	Group 2 (*n* = 24)	OR	95% CI		Group 1 (*n* = 3)	Group 2 (*n* = 27)	OR	95% CI	
Age of ≥90 yrs	10 (55.6)	3 (25.0)	3.75	0.75–18.6	0.14	3 (50.0)	10 (41.7)	1.4	0.23–8.42	1.00	2 (66.7)	11 (40.7)	2.91	0.23–36.2	0.56
Male sex	3 (16.7)	2 (16.7)	1.00	0.14–7.10	1.00 (0.76)^*d*^	1 (16.7)	4 (16.7)	1.00	0.09–11.0	1.00 (0.66)^*d*^	0 (0.0)	5 (18.5)	0		1.00
PS score = 4	15 (83.3)	11 (91.7)	0.45	0.04–4.98	0.63	6 (100.0)	20 (83.3)			0.56	3 (100.0)	23 (85.2)			1.00
With remaining teeth	15 (83.3)	8 (66.7)	2.50	0.45–14.0	0.39	5 (83.3)	18 (75.0)	1.67	0.16–17.3	1.00	2 (66.7)	21 (77.8)	0.57	0.04–7.44	1.00
OHAT-J score															
Lip score = 1	6 (33.3)	2 (16.7)	2.5	0.41–15.2	0.42	3 (50.0)	5 (20.8)	3.8	0.58–24.9	0.3	1 (33.3)	7 (25.9)	1.43	0.11–18.3	1.00
Tongue score = 1 or 2	6 (33.3)	4 (33.3)	1.00	0.21–4.71	1.00	1 (16.7)	9 (37.5)	0.33	0.03–3.33	0.63	0 (0.0)	10 (37.0)			0.53
Gum and mucosa score = 1 or 2	6 (33.3)	3 (25.0)	1.5	0.29–7.68	0.70	2 (33.3)	7 (29.2)	1.21	0.18–8.22	1.00	0 (0.0)	9 (33.3)			0.53
Saliva score = 1 or 2	10 (55.6)	3 (25.0)	3.75	0.75–18.6	0.14	3 (50.0)	10 (41.7)	1.4	0.23–8.42	1.00	1 (33.3)	12 (44.4)	0.63	0.05–7.75	1.00
Natural teeth score = 1 or 2	8 (44.4)	3 (25.0)	2.4	0.48–11.9	0.44	2 (33.3)	9 (37.5)	0.83	0.13–5.50	1.00	1 (33.3)	10 (37.0)	0.85	0.07–10.6	1.00
Denture score = 1	1 (5.6)	0 (0)			1.00	0 (0.0)	1 (4.2)			1.00	0 (0.0)	1 (3.7)			1.00
Oral cleanliness score = 1 or 2	7 (38.9)	4 (33.3)	1.27	0.28–5.87	1.00	1 (16.7)	10 (41.7)	0.28	0.03–2.78	0.37	0 (0.0)	11 (40.7)			0.28
Tooth pain score = 1	0 (0.0)	1 (8.3)			0.40	0 (0.0)	1 (4.2)			1.00	0 (0.0)	1 (3.7)			1.00
Situation before admission															
From home	3 (16.7)	1 (8.3)	2.20	0.20–24.1	0.63	0 (0.0)	4 (16.7)	0		0.56	0 (0.0)	4 (14.8)	0		1.00
From another hospital	6 (33.3)	1 (8.3)	5.5	0.57–53.2	0.19	3 (50.0)	4 (16.7)	5.00	0.73–34.3	0.12	1 (33.3)	6 (22.2)	1.26	0.12–13.6	1.00
From affiliated hospital	4 (22.2)	3 (25.0)	0.86	0.15–4.76	1.00	1 (16.7)	6 (25.0)	0.6	0.06–6.21	1.00	0 (0.0)	7 (25.9)	0		1.00
From another facility	5 (27.8)	5 (41.7)	0.54	0.12–2.52	0.46	1 (16.7)	9 (37.5)	0.33	0.03–3.33	0.63	1 (33.3)	9 (33.3)	1.00	0.08–12.6	1.00
History of medical visits within 1 mo	1 (5.6)	2 (16.7)	0.29	0.02–3.67	0.55	0 (0.0)	3 (12.5)			1.00	0 (0.0)	3 (11.1)			1.00
Prior use of antibiotics within 6 mo	3 (16.7)	7 (58.3)	0.14	0.03–0.77	0.045* (0.024*)^*d*^	2 (33.3)	8 (33.3)	1.00	0.15–6.67	1.00	0 (0.0)	10 (37.0)	0		0.53
Tube feeding	7 (38.9)	3 (25.0)	1.91	0.38–9.59	0.69	5 (83.3)	5 (20.8)	19	1.79–201	0.0088** (0.015*)^*d*^	2 (66.7)	8 (29.6)	4.75	0.38–60.1	0.25
Nasogastric tube	3 (16.7)	1 (8.3)				2 (33.3)	2 (8.3)				1 (33.3)	3 (11.1)			
Gastrostomy and enterostomy	4 (22.2)	2 (16.7)				3 (50.0)	3 (12.5)				1 (33.3)	5 (18.5)			
Presence of comorbidities															
Strokes	9 (50.0)	5 (41.7)	1.40	0.32–6.11	0.72	5 (83.3)	9 (37.5)	8.33	0.84–83.2	0.07	2 (66.7)	12 (44.4)	2.5	0.20–31.0	0.59
Cardiovascular disease	7 (38.9)	4 (33.3)	1.27	0.28–5.87	1.00	4 (66.7)	7 (29.2)	4.86	0.72–32.9	0.16	2 (66.7)	9 (33.3)	4.00	0.32–50.2	0.54
Diabetes	3 (16.7)	0 (0)			0.26	0 (0.0)	3 (12.5)	0		1.00	0 (0.0)	3 (11.1)	0		1.00
Tumor bearing	2 (11.1)	2 (16.7)	0.63	0.08–5.17	1.00	1 (16.7)	3 (12.5)	1.4	0.12–16.5	1.00	0 (0.0)	4 (14.8)	0		1.00

a OR, odds ratio; CI, confidence interval. *, *P* value of ≤0.05; **, *P* value of ≤0.01.

b Group 1, subjects with oral isolates that showed resistance or intermediate resistance; group 2, subjects without oral isolates that showed resistance or intermediate resistance.

c By Fisher’s exact test.

d By multiple logistic regression analysis.

We also used another criterion based on the results of our analysis using MIC_50_ and MIC_90_, as shown in Table S5. CPC resistance or intermediate resistance was significantly related to tube feeding (*P* = 0.0048), while other recorded variables were not related to CPC resistance. BZK resistance or intermediate resistance showed a significant relationship with an Oral Health Assessment Tool—Japanese edition (OHAT-J) oral cleaning status score of 1 or 2 (*P* = 0.021) and the presence of cardiac disease (*P* = 0.021). An OHAT-J score of 1 or 2 indicates poor oral hygiene. No factors were significantly associated with CHX resistance or intermediate resistance. Furthermore, to confirm whether these items were associated with disinfectant resistance regardless of other factors, we performed multiple logistic regression analysis on each item after adjusting for covariates; this analysis showed that resistance to CPC was associated with tube feeding (OR, 16.1 [95% CI, 2.19 to 119]; *P* = 0.0063).

## DISCUSSION

In this study, we first evaluated the susceptibilities of oral and rectal GN-ARB from LTCF residents to 4 disinfectants that are often applied to the oral cavity. In a comparison of the susceptibility of bacterial genera to disinfectants, Acinetobacter tended to be susceptible to disinfectants, although A. nosocomialis showed less susceptibility to CHX than other Acinetobacter species. Among Pseudomonas species, P. aeruginosa showed a high degree of resistance to CPC and BZK, and some P. aeruginosa isolates also showed a high degree of resistance to CHX, while other Pseudomonas species isolates showed susceptibility to these disinfectants. Most E. coli and Enterobacter species isolates were highly susceptible to all disinfectants. S. A. Hammond et al. reported that P. aeruginosa showed 3- to 5-times-higher MIC values of BZK than *P. cepacia* (15). Köhler et al. reported that most A. baumannii strains were susceptible to BZK, while P. aeruginosa strains showed the lowest susceptibility among multidrug-resistant Acinetobacter, Pseudomonas, and Klebsiella strains (16). In addition, it was reported that P. mirabilis exhibited a lower level of susceptibility to CHX than other species such as A. baumannii, E. coli, and P. aeruginosa (15, 17). The results of our analysis of susceptibility to disinfectants among several bacterial species showed trends that were similar to those observed in previous studies of isolates from various sites such as the rectum and blood. In our comparison of the MIC values of the 4 disinfectants with the concentrations used in the oral cavity, the MICs of CPC and BZK were higher than the concentrations used in the oral cavity. Although the CHX concentration used in the oral cavity is generally higher than the MICs for common bacteria, the concentration used in Japan is significantly almost 10-fold lower (18, 19), revealing that some isolates have especially high CHX MIC values in Japan. Based on our results in this study, we found that some oral GN-ARB showed not only antibiotic resistance but also disinfectant resistance.

In the isolates that were identified to exhibit the same oral and rectal ST when obtained from the same person, we performed SNP analysis and found that the number of SNPs was relatively low. Therefore, we can infer that the oral and rectal isolates obtained from these six persons are the same clones within individual hosts. Additionally, as there were some SNPs in all isolates from oral and rectal samples of the same persons, we speculated that the mutations occurred during the localization of the isolate to different environments. Since the participants were LTCF residents requiring nursing care (or support) for cognitive decline, we speculate that rectally derived bacteria may have entered some participants’ oral cavity by some route; one possibility is that bacteria directly migrated into the oral cavity from the patients’ hands after direct contact with the rectum, and the other is that bacteria migrated into the oral cavity from objects in the environment that had been contaminated by the patients’ rectum, such as the bed, linens, and caregivers or other staff members.

We found a correlation between *qacE*Δ*1* and QAC resistance in P. aeruginosa. However, P. aeruginosa isolates without *qacE*Δ*1* showed higher MIC values than other bacterial species. In P. aeruginosa, multidrug pumps of the resistance-nodulation-division (RND) superfamily that are known as Mex pumps, such as MexAB-OprM and MexCD-OprJ, have been demonstrated to be involved in susceptibility to QACs and CHX (20, 21). All P. aeruginosa isolates used in this study possessed both genes, which suggests that these two factors were also involved in resistance to QACs. In addition, a previous study reported that ST235 isolates of P. aeruginosa produced large amounts of biofilm (2); thus, it is possible that this ability is related to disinfectant susceptibility.

In E. coli, the existence of *qacE*Δ*1* did not show a relationship with resistance to QACs. The *mdfA*, *sugE(c)*, and *ydgEF* genes were found in all E. coli isolates, but we did not find a relationship between these genes and resistance to QACs because all E. coli isolates tested showed low MIC values for QACs and chlorhexidine. Zou et al. reported that *mdfA-sugE(c)-ydgEF* was the most common resistance genotype in E. coli, but they found a significant association between QAC resistance and the existence of *qacE*Δ*1* and/or *sugE* (22). Previously, it was demonstrated that the introduction of several genes responsible for disinfectant susceptibility, including *qacE*Δ*1*, into disinfectant-susceptible E. coli isolates did not result in drastic resistance to QACs (23, 24). However, in light of our results, this factor might not be associated with QAC resistance in the isolates used. Based on the results of previous reports in addition to our results, we conclude that even the presence of resistance genes, including *qacE*Δ*1*, did not always result in QAC and chlorhexidine resistance in E. coli isolates. Similar to E. coli isolates, 5 *qacE*Δ*1*-positive P. mirabilis isolates from the oral cavity in this study did not show low susceptibility to QACs, similar to *qacE*Δ*1*-positive P. aeruginosa, although the MIC value of QACs in P. mirabilis was higher than that in E. coli. In contrast, these P. mirabilis isolates showed resistance to chlorhexidine. H. Pelling et al. reported that the *smvA* efflux system was involved in susceptibility to biocides, including chlorhexidine and QACs (25). Additionally, the *smvA* gene was also found in some *Enterobacteriaceae* species and was demonstrated to be associated with biocide susceptibility (26). Therefore, *smvA* in P. mirabilis might contribute to resistance to CHX and low susceptibility to QACs.

In this study, we found that *qacE*Δ*1* was located in class 1 integrons of several bacterial species (Fig. 2). Integrons are known to be multiple-gene acquisition systems located in the bacterial genome, and class 1 integrons are found extensively in clinical isolates (27). Many antibiotic resistance gene cassettes that include disinfectant resistance genes were found in class 1 integrons (27). In P. aeruginosa, *qacE*Δ*1* and aminoglycoside resistance genes (*aadB*, *aadA6*, *aacA4*, and *aacA5*) were most commonly found (28). In addition, many genes encoding β-lactamases, including the *bla*_IMP-1_, *bla*_VIM-2_, *bla*_OXA-10_, and *bla*_VIM-1_ genes, were found in the class 1 integron cassettes of some P. aeruginosa strains (28–30). The integron *intI1-bla*_IMP-11_*-aacA1-orfG-qacE*Δ*1-sul1* was found in E. coli and K. pneumoniae (31). Therefore, the integron 1 cassette provides resistance to multiple antibiotic compounds. In this study, we found *qacE*Δ*1*, *sul1*, and an aminoglycoside resistance gene in the integron 1 cassettes of some isolates, but we did not find the genes coding for β-lactamase and carbapenemase (Fig. 2). We identified 7 ESBL-positive isolates, including 4 E. coli, 1 *E. hormaechei*, and 2 P. mirabilis isolates, but the genes coding for ESBL were not found in the integron 1 cassette.

In our analysis of patient risk factors, we included items to evaluate oral hygiene status and the number of teeth because we focused on oral GN-ARB. We used two criteria for the classification of susceptibility. One criterion was defined by using the concentration for oral application, and the other criterion was defined by using the MIC_50_ of each disinfectant in oral isolates (Table 5; see also Table S5 in the supplemental material). The analysis showed an association between CPC resistance and tube feeding (Table 5; Table S5). Kajihara et al. reported a correlation between tube feeding and the presence of ESBL-producing *Enterobacterales* or P. aeruginosa in the oral cavity and rectum (2). In addition, an interesting correlation was found between the presence of disinfectant-resistant bacteria in the oral cavity and tube feeding. Although the reason for this correlation is not clear, it may be because hospitalized patients receiving tube feeding are often exposed to drug- and disinfectant-resistant organisms. Aspiration pneumonia is the most common cause of death in patients fed with gastrostomy tubes (3). Based on these results, we need to take greater care to sterilize tubes with appropriate disinfectants to prevent infectious diseases.

In conclusion, we found that some third-generation-cephalosporin- or carbapenem-resistant Gram-negative bacteria isolated from the oral cavity of residents of LCTFs were resistant to some disinfectants. Also, we found an association of low susceptibility to disinfectants with the existence of previously identified disinfectant resistance genes in some isolates, but many isolates showed no association, suggesting the presence of other disinfectant resistance factors. Furthermore, the rate of isolation of disinfectant-resistant bacteria was significantly higher in patients on tube feeding. In LTCFs, routine oral care using mouthwashes is sometimes performed. This suggests that further consideration should be given to the use of mouthwashes containing disinfectants, especially for the elderly and pre- and postoperative patients.

## MATERIALS AND METHODS

### Bacterial strains.

The clinical isolates used in this study are listed in Table 1. Among the isolates, 32 oral isolates and 48 rectal isolates were previously isolated using CHROMagar ESBL medium plates (Kanto Chemical, Tokyo, Japan) as cephalosporin-resistant isolates (2). Twenty oral isolates and 27 rectal isolates obtained by using CHROMagar mSuperCARBA medium plates (Kanto Chemical, Japan) were used (see Fig. S1 and Table S6 in the supplemental material). We also used standard strains, including 2 E. coli strains (K-12 and ATCC 25922), 2 P. aeruginosa strains (PAO1 and ATCC 27853), 1 K. pneumoniae strain (ATCC BAA-1706), 2 A. baumannii strains (ATCC 19606 and ATCC 17978), and 1 A. pittii strain (ATCC 19004). Each strain was cultured in LB broth at 37°C under aerobic conditions.

### Clinical data.

Clinical information from 30 participants was obtained previously (Hiroshima University Hospital review board approval number E-1692). We excluded eight participants who had oral GN-ARB because we could not collect information from one facility. The information that we collected included demographics (age, sex, and unit of residence), Eastern Cooperative Oncology Group (ECOG) performance status (PS), situation before admission, history of medical visits, use of antibiotics within the prior 6 months, nutrition type, and presence of comorbidities. The OHAT-J was used to assess oral health status (32). The OHAT-J is an oral screening tool that allows nursing and caregiving staff to easily evaluate the oral condition of persons requiring nursing care, and high reliability and validity between the OHAT-J and the original version of the OHAT developed by Chalmers et al. have been reported (32, 33). This method is performed by visual examination of the lips, tongue, gingiva, mucosa, saliva, remaining teeth, oral cleaning status, toothache, and denture fracture and fit. Each item is rated on a scale from 0 to 2. High scores indicate poor oral hygiene (https://www.ohcw-tmd.com/research).

### MIC determination.

The MIC was determined by the microdilution method as described previously (34). The disinfectants used in this study were povidone iodide (PVPI; Mundipharma KK, Tokyo, Japan), cetylpyridinium chloride (CPC; Fujifilm Wako Pure Chemical Corporation, Osaka, Japan), benzalkonium chloride (BZK; Fujifilm Wako Pure Chemical Corporation), and chlorhexidine chloride (CHX; Fujifilm Wako Pure Chemical Corporation). Twofold serial dilutions of each disinfectant were prepared in 96-well plates (Thermo Fisher Scientific, Waltham, MA, USA). The maximum concentrations of PVPI, CPC, BZK, and CHX were 35,000, 5,120, 640, and 8,192 μg/mL, respectively. A culture of each bacterium grown overnight was adjusted to an optical density at 660 nm (OD_660_) value of 1.0 (1 × 10^9^ cells/mL), and the culture was then diluted 100-fold with tryptic soy broth (TSB) (1 × 10^7^ cells/mL). Ten microliters of the dilution was inoculated into each well (100 μL). After 24 h of aerobic incubation at 37°C, MICs were determined.

We defined the criterion based on the concentration of each disinfectant used for mouthwash (PVPI, 2,188 to 4,375 μg/mL [0.12 to 0.23%] [35, 36]; CPC, 400 to 800 μg/mL [0.05 to 0.1%] [37–41]; BZK, 80 to 800 μg/mL [0.01 to 0.1%] [41, 42]; CHX, 1,299 to 2,048 μg/mL [0.12 to 0.2%] [40, 43–45] [Table S2]). The criteria for susceptibility (S), intermediate resistance (I), and resistance (R) were defined as follows: PVPI, S at <2,188 μg/mL and R at ≥4,375 μg/mL; CPC, S at <400 μg/mL, I at ≥400 μg/mL and <800 μg/mL, and R at ≥800 μg/mL; BZK, S at <80 μg/mL, I at ≥80 μg/mL and <800 μg/mL, and R at ≥800 μg/mL; and CHX, S at <1,299 μg/mL, I at ≥1,299 μg/mL and <2.048 μg/mL, and R at ≥2,048 μg/mL.

Additionally, we defined another criterion for R, I, and S, as follows: R at greater than or equal to the MIC_90_, I at less than the MIC_90_ and greater than the MIC_50_, and S at less than or equal to the MIC_50_ (Table S7). Antimicrobial susceptibility was determined using the Walkaway system (Beckman Coulter, USA) as described in a previous study (2).

### Genetic analysis.

We chose to analyze major genes responsible for CPC, BZK, and CHX resistance in E. coli, Enterobacter, Acinetobacter, Pseudomonas, and Proteus. For BZK/CPC and CHX resistance, *qacE*Δ*1*, *qacA–J*, *mdfA*, *sugE(c)*, *ydgE*, *ydgF*, *emrE*, *smvA*, *cepA*, *mexAB-oprM*, and *mexCD-oprJ*, which were previously reported, were selected (15–17, 20, 21). Genome sequencing was conducted as described previously (2). For the 47 isolates newly collected in this study, genome sequencing was conducted. SnapGene software (www.snapgene.com) was used to detect disinfectant resistance genes and ESBL genes from whole-genome sequencing data. We also analyzed the disinfectant genes and ESBL genes with ResFinder (Center for Genomic Epidemiology [https://cge.food.dtu.dk/services/ResFinder/]) (46). Based on the genome data for each isolate, multilocus sequence typing (MLST) and phylogenetic tree analysis were performed. Phylogenetic trees were generated by using the CSI Phylogeny 1.4 pipeline available from the Center for Genomic Epidemiology (Lyngby, Denmark). Next, the tree was annotated using Interactive Tree of Life (iTOL) software.

SNP analysis was performed by mapping the Illumina reads of two isolates using progressive Mauve (47). The SNPs in the region within 1,000 bp from the terminus of each contig were removed due to a high tendency toward error in these regions.

### Statistics.

The correlation between clinical information and oral disinfectant-resistant bacteria was analyzed by Fisher’s exact test. Multiple logistic regression analysis was performed for age, sex, and factors for which the analysis revealed *P* values of less than 0.05 upon univariate analysis. Additionally, we used the Wilcoxon rank sum test to analyze the relationship between the presence of *qacE*Δ*1* and disinfectant susceptibility.

The results with a *P* value of less than 0.05 were considered significant for all statistics. All statistical analyses were conducted using JMP Pro version 16 (SAS Institute, Cary, NC, USA).

### Ethics.

All residents admitted to LTCFs during the study period were eligible for inclusion. Written informed consent was obtained from the participants prior to their enrollment in the study. Additionally, we obtained written informed consent from the families of participants who lacked the mental capacity to consent. The residents were excluded if they or their families refused consent. This study was approved by the ethical committees of the Hiroshima University Hospital review board (approval number E-1692) and the National Institute of Infectious Diseases Committee of Ethics (approval number 1017). All study protocols were performed in accordance with the principles of the Declaration of Helsinki.

### Data availability.

The genome data for the isolates used in this study have been deposited in the NCBI database (BioProject accession no. PRJDB14068 and PRJDB12075) (2).
