# Supplementary figures and images for: Disinfectant Susceptibility of Third-Generation-Cephalosporin/Carbapenem-Resistant Gram-Negative Bacteria Isolated from the Oral Cavity of Residents of Long-Term-Care Facilities
Source: Appl Environ Microbiol. 2022 Dec 14;89(1):e01712-22. doi: 10.1128/aem.01712-22 (PMC9888285; doi:10.1128/aem.01712-22)

**178 long-term care facility residents**

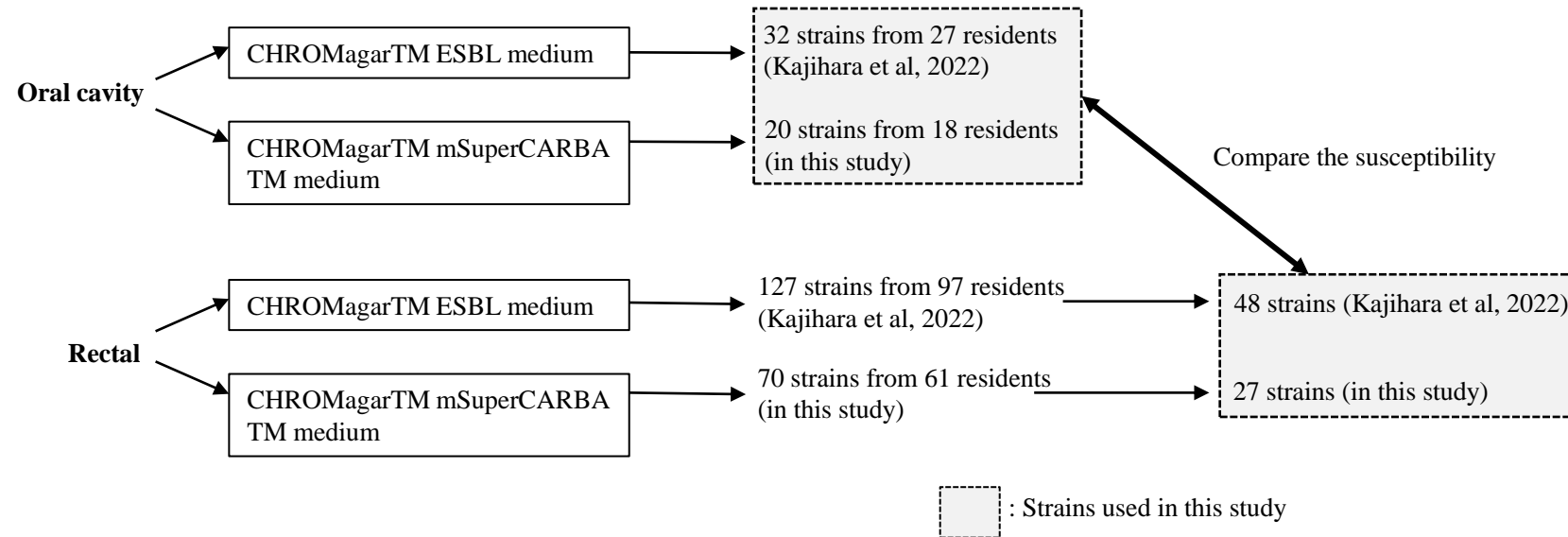

Supplemental Fig. 1. Isolates used in this study

Supplement: Supplemental file 8 — Fig. S1. Download aem.01712-22-s0008.pdf, PDF file, 0.01 MB [file aem.01712-22-s0008.pdf]
